# Mapping of Cu and Pb Contaminations in Soil Using Combined Geochemistry, Topography, and Remote Sensing: A Case Study in the Le’an River Floodplain, China

**DOI:** 10.3390/ijerph9051874

**Published:** 2012-05-16

**Authors:** Yiyun Chen, Yaolin Liu, Yanfang Liu, Aiwen Lin, Xuesong Kong, Dianfeng Liu, Xiran Li, Yang Zhang, Yin Gao, Dun Wang

**Affiliations:** 1 School of Resource and Environmental Science, Wuhan University, Luoyu Road 129, Wuhan 430079, China; Email: kellypcyy@126.com (Y.C.); yfliu610@163.com (Y.L.); awlin@263.net (A.L.); kxsiu@tom.com (X.K.); liudfeng1985@126.com (D.L.); lxrna@163.com (X.L.); 252070680@qq.com (Y.Z.); 494141963@qq.com (Y.G.); 12021715@qq.com (D.W.); 2 Key Laboratory of Geographical Information System, Ministry of Education, Wuhan University, Luoyu Road 129, Wuhan 430079, China

**Keywords:** soil, heavy metal, contamination, digital elevation model, mapping

## Abstract

Heavy metal pollution in soil is becoming a widely concerning environmental problem in China. The aim of this study is to integrate multiple sources of data, namely total Cu and Pb contents, digital elevation model (DEM) data, remote sensing image and interpreted land-use data, for mapping the spatial distribution of total Cu and Pb contamination in top soil along the Le’an River and its branches. Combined with geographical analyses and watershed delineation, the source and transportation route of pollutants are identified. Regions at high risk of Cu or Pb pollution are suggested. Results reveal that topography is the major factor that controls the spatial distribution of Cu and Pb. Watershed delineation shows evidence that the streamflow resulting from rainfall is the major carrier of metal pollutants.

## 1. Introduction

Highly accumulated heavy metals in soil could be harmful to human beings from either direct exposure or via food chains [1]. Mapping the heavy metal distribution around and downstream from mining areas is important because better mapping usually means better identification of pollution sources and better understanding of the transportation and settlement of pollutants. When mapping the heavy metal contaminations in soil, two aspects have been considered in relatively few previous studies: the land use and the topography of the study area.

One basic rule of human health risk assessment (HHRA), which is one of the most important tools for the assessment of soil contamination [2], is that the risk of heavy metal pollutants to human health is land-use dependent [3,4]. It is because the land-use types and functions decide the exposure pathway and types of potential risks from contaminations [3]. Thus, it is essential to consider the land-use when mapping the heavy metal contaminations in soil. In areas where the land-use map is unavailable, remote sensing image holds the potential and efficiency in land use interpretation. Moreover, due to its approximately true color representation of the real world, remote sensing image can serve as a background map when mapping soil contaminations.

Topography is also an important aspect that must be taken into account, especially when mapping heavy metal pollution from mining and smelting activities in mountain areas. As a major source of environmental pollution, mine tailings is scoured by precipitation, and streams carrying heavy metal pollutants thereby converge into larger rivers or penetrate into the earth. In this process, the topography is the major factor that controls the water flow paths [5].

Therefore, combined with geochemistry, topography, and remote sensing, this study aimed to map the total copper and lead contents in top soil within a buffer zone of 200 m along the Le’an River and its branches. Based on the resulting maps, the sources, extents and potential risks of the heavy metal pollution are explored. The results of this study may promote our understanding of the roles that topography and land use play in soil pollution in a mining area and its downstream regions.

## 2. Materials and Methods

### 2.1. Description of the Study Area

**Figure 1:**
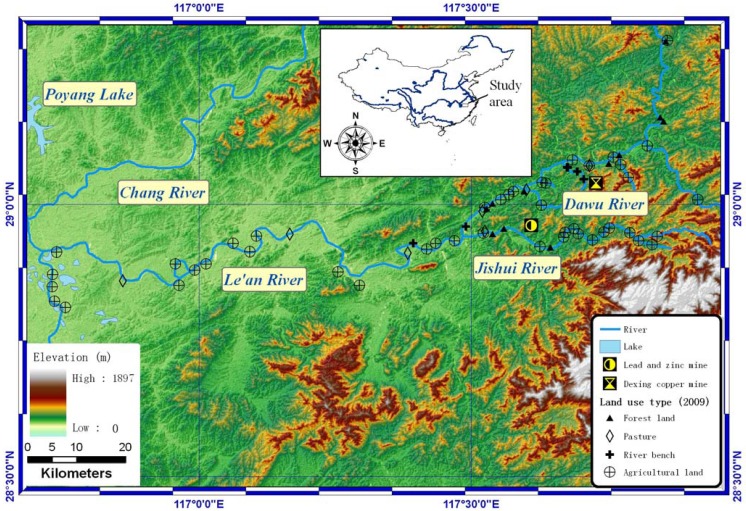
Map showing Poyang Lake, Le’an River, Jishui River, Dawu River, Yishan lead-zinc mine, Dexing copper mine, sampling sites and elevation of the study area.

A case study was conducted in the Le’an River floodplain, Jiangxi Province, China (28.7–29.3° N and 116.5–117.9° E, Figure 1). In the study area, the Le’an River runs 279 km from east to west into Poyang Lake, which is the largest freshwater lake in China. In the west of this region, the topography is relatively flat with elevation around 0–200 m. Several counties surrounded by large scale agricultural land locate along the Le’an River. For the mountain area in the east, the elevation is around 500–1,900 m with relatively ridged topography. This area covers several mining regions, including the Dexing copper mine and the Yishan lead-zinc mine [6]. The Dexing copper mine is the largest outcrop copper mine in China. The Dawu River crosses this mining area and drains into the Le’an River [7]. The Yishan lead-zinc mine locates downstream of the Jishui River, which is the largest branch of the Le’an River [6].

### 2.2. Soil Sample Collection and Chemical Analyses

A total of 71 top layer (0–15 cm) soil samples were collected in the Le’an River floodplain from 29 October 2009 to 1 November 2009. Most of the sample sites were close to the Le’an River and its branches (<50 m). About 75% of the samples (53 samples) were collected in the middle and upper reaches of the Le’an River where the Dexing copper mine and Yinshan lead-zinc extraction facility are located (Figure 1). Four portions of topsoil samples (0–15 cm) within a 10 m^2^ plot were collected and mixed at each sample site. Stones and debris were removed. Approximately 1 kg soil sample was stored in a plastic bag, which was numbered with a tag. The geographical coordinates were also recorded by a handheld global position system (GPS) with a positional error <10 m. All soil samples were taken to the laboratory on the fourth day of the fieldwork.

In laboratory, all the 71 soil samples were first air-dried at 20–25 °C for two days. The dried soil samples were then gently crushed in a porcelain mortar to break up large aggregates and sieved using a 0.2 mm stainless steel sieve. Total contents of Cu and Pb were determined by wavelength-dispersive X-ray fluorescence (XRF) spectroscopy (X–50TM Mobile XRF Analyzer, Boston, MA, USA). All the samples were homogenized before XRF analysis. An elastic plastic collar was placed on a stainless steel plate, and then about 40 g of each sample was filled in and afterwards compressed by a semi-automatic press machine into a uniform pellet of 45 mm diameter. The XRF analyzer is advantageous for its simultaneous estimation of several metal elements, without producing much chemical wastes.

### 2.3. Statistical Analyses

Statistical analyses, including descriptive statistics, histograms, normal probability plots, Q-Q plots (normal quantile-quantile plots) and the Lilliefors normality test were adopted in this study and implemented in Matlab^®^ (R2008a). Descriptive statistics was used to describe the main features of the total Cu and Pb contents of the collected soil samples. The histograms could visually depict the data distributions of the total Cu and Pb contents. Normal probability plots and Q-Q plots were used to graphically assess the data normality of the total Cu and Pb contents, while the Lilliefors normality test was used to test the null hypothesis that data come from a normally distributed population, when the null hypothesis does not specify which normal distribution.

### 2.4. Watershed Delineation

The watersheds of the Jishui River and Dawu River were generated from GDEM data using the Hydrology Toolbox in ArcGIS. The first step was to fill sinks, which were areas of internal drainages. With the filled DEM data, maps of flow direction and accumulation could be consequently generated. The outlet or pour points were assigned to those locations which were both downstream of the Jishui River or Dawu River, and close to sample sites. Thus, the boundary of the watershed draining to the pour point could be identified.

### 2.5. Flowchart

**Figure 2:**
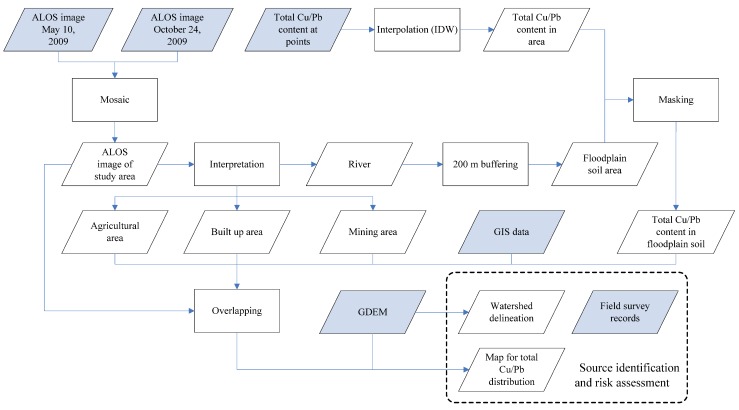
Flowchart of mapping total Cu and Pb contaminations.

The flowchart for mapping the spatial distribution of heavy metal content is shown in Figure 2. It presents the data used (parallelogram with no color filled), the process (square) and the data or results derived (filled parallelogram). Two compositive maps showing spatial distributions of total Cu and Pb contaminations in the top soil along the Le’an River are the expected outcomes. Further analyses are thereafter based on these two maps, watershed delineation and field survey records. The essentiality of these data shown in Figure 2 and the roles they act are further illustrated in the followings.

**Figure 3:**
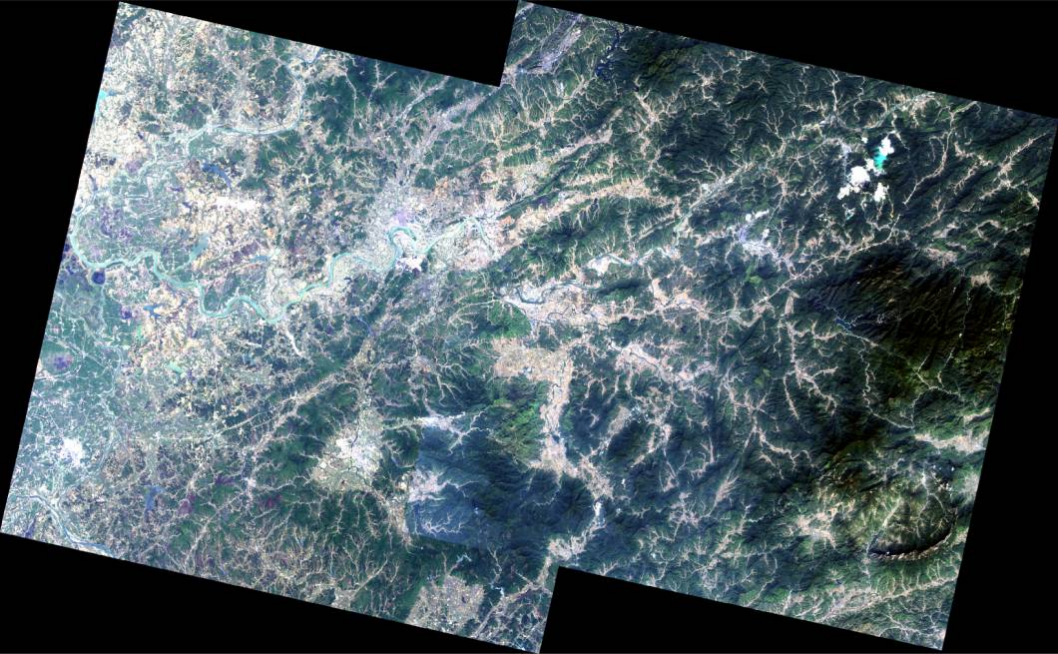
Two ALOS images showing the study area.

Two ALOS AVNIR-2 (Advanced Visible and Near-Infrared Radiometer type 2) images at 1B2 level (with both radiometric and geometric correction) were used to cover the whole study area (Figure 3). The left was taken on 10 May 2009, and the right was captured on 24 October 2009, which was five days before field sampling. In the area where they are overlapping, the later one was used in the “Mosaic” process. A true color image of the study area was generated with R, G and B corresponding to Band 3 (0.61–0.69 μm), Band 2 (0.52–0.60 μm) and Band 1 (0.42–0.50 μm), respectively [8]. This image was used both for interpreting the land use types and as the background map of the final compositive maps. Three land use types were derived from the image: agricultural area, built up area and mining area. Rivers in the study area were also identified from the same image.

With rivers buffer zones (200 meters) calculated from the rivers, the extents of floodplain soil area where most soil samples of this study were taken can thereby be sketched. The ASTER Global Digital Elevation Map (GDEM) data, which showed the topography of the study area, was used for building the 3D terrain model and for flow accumulation analyses. The total Cu and Pb contents at sampling sites were used for inverse distance weighted (IDW) interpolation, which could offer a rough estimation of heavy metal content in areas without measurement. As most of the sample sites were within a 200 m buffer zone of rivers, and more prone to the influences of the water flows, the interpolation results were less reliable outsides the buffer zone. Thus, the extents of the floodplain soil area were used as a mask, within which the interpolated results of total Cu and Pb contents were retained and displayed. In the final step, the ALOS image, the maps of agricultural area, built up area and mining area, total Cu/Pb content in floodplain soil area, and GIS data such as administrative boundary and location of cities were overlapped to generate a 2D map. All the above analyses were processed in ArcGIS. In order to map all these elements in a three dimension, the ArcScene software was used. The ASTER GDEM data was used as base height. The sun azimuth and altitude at the time when the right image was taken were 126 degrees and 31 degrees (the center point of the image), respectively. Thus, the azimuth and altitude of the simulated sunlight of the 3D map were respectively set to 126 degrees and 31 degrees.

## 3. Results and Discussion

### 3.1. Sample Characterization

Summary statistics for total Cu and Pb contents are provided in Table 1. In the study area, the estimated concentrations of Cu were much higher than the average Chinese soil background value of 20.7 mg/kg [9]. 

**Table 1:** Characteristics of total Cu and Pb contents in the soil sample set (n = 71).

Heavy metal	Min	Max	Mean	Median	Standard deviation	China soil background value [9]
Total Cu content (mg/kg)	90	3,034	290	126	470	20.7
Total Pb content (mg/kg)	29	605	90	74	82	23.5

The value was also higher than the suggested local background value (48 mg/kg for non-agricultural top soil and 51 mg/kg for agricultural top soil) from a contemporary geochemical survey in the Dexing area, where 53 samples of our study were collected [10]. The distribution of the sampling sites might explain part of this difference as most sample sites in our study were located along the river, and therefore easily influenced by floods carrying heavy metals. Study carried out in nonferric metal mining area in surface soils in some countries reported comparable copper content, e.g., in Japan (456–2,020 mg/kg) [11]. The lead content (Table 1) was below the average contamination level in nonferric metal mining area in the Great Britain (170–4,563 mg/kg) [12], the U.S. (15–13,000 mg/kg) [13], and the Germany (>300 mg/kg) [14].

**Figure 4:**
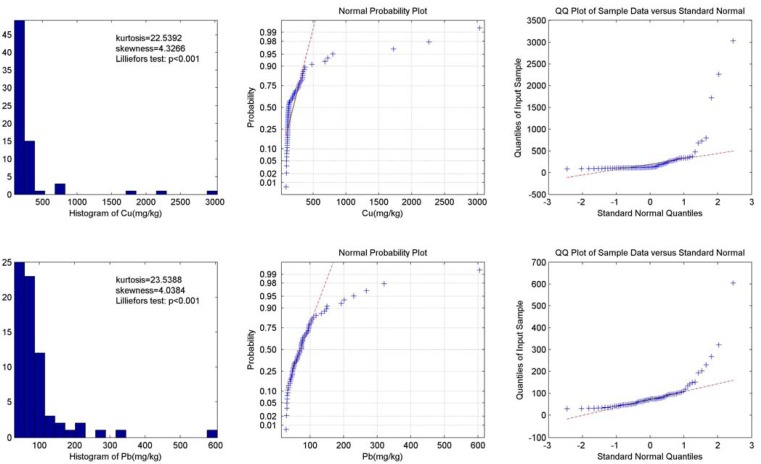
Data distribution and normality test results of total Cu and Pb content.

The histogram, normal probability plot and Q-Q plot of total Cu and Pb contents are shown in Figure 4. Graphical inspections of both the total Cu and Pb contents show that the data is highly positively skewed (skewness = 4.33 and 4.04, respectively) and asymmetrical with a steep peak (kurtosis = 22.54 and 23.54, respectively). The Lilliefors normality test, normal probability plot and Q-Q plot show evidence of non-normality. The histogram also suggests that there are some samples with extreme values. Therefore, the Krigking interpolation strategies were not used in this study for their normality assumption.

### 3.2. Mapping the Total Cu and Pb Contents

Two series of maps derived from an integration of multiple data sources are presented below to show the spatial distribution pattern of total Cu and Pb in the soil along the Le’an River. In these maps, color scales from blue for low concentration of Cu/Pb to red for high Cu/Pb content in soil. Sample sites and cities are also marked with 3D symbols. The estimation of heavy metal contents in area is calculated from point interpolation. Thus, the estimation could be generally more reliable in the area which is closer to sample sites. Interpretation results from Alos images shows the built up area (in grey), agricultural area (in light green), and mining area (in orange). The transparency of the land use type layer was set to 50% for a better visual inspection.

### 3.3. Source Identification and Analysis

**Figure 5:**
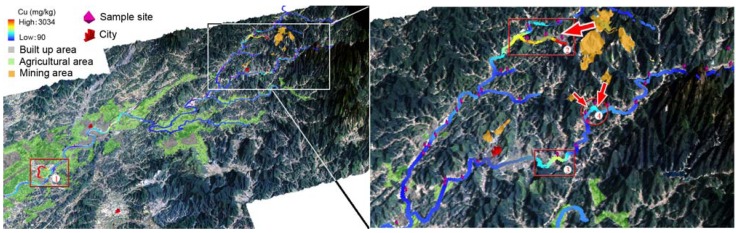
Maps showing the distribution of total Cu content in the soil along the Le’an River and its branches (left), and in the soil around mining area (right).

The overall spatial distributions of the total Cu content in the top soil along the Le’an River are shown in the left of Figure 5. An area with high total Cu content is highlighted by a red rectangle and noted as “①”. The soil sample in this area was taken from the river terrace of a short tributary downstream of the Le’an River in the Raobu County, which has an estimated population of 49 thousands and cultivated land of 20 km^2^. The total Cu content reached as high as 2262 mg/kg. An explanation for such a high value could be that the water velocity in this tributary was low. Thus, suspended sediment which was rich in Cu content deposited here. This hypothesis needs to be confirmed with more soil samples collected around this area and with the water velocity measured.

The total Cu content distributions around the Dexing Copper Mine are shown in the right of Figure 5. Three regions, at least, were rich in total Cu content. They were respectively noted as “②”, “③”, and “④” in the figure. 

Soil samples from the terrace of the region ② (red square), which located at the middle and lower reaches of the Dawu River, showed up with an average of Cu content at about 800 mg/kg. The mining area highlighted in light orange could be the source. The potential transportation route of Cu was suggested with a red arrow. The source and route was thereafter confirmed by flow accumulation analyses. Little agricultural land was found in this area. Thus, the risk of Cu up taken by local residents through food chain could be low. According to our survey, however, significant atmospheric dust arose from the mechanical disturbance. Particulate matters (e.g., PM_2.5_) from road dust resulting from vehicular traffic needed to be monitored with regular reports published. The use of watering cart (which had been witnessed in this survey) and breathing mask could be helpful for keeping the residents here from potential exposure to pollutants carried by aerosol.

Regions ③ and ④ are located in the middle and lower reaches of the Jishui River (Figure 5). Possible transportation of total Cu content was marked by red arrows through visual inspections (Figure 5). With consideration of the topography and the precipitation, the soil downhill in the region ④ might be polluted by pollutants from two mine tailings uphill. A small town named Xinying located in the regions ③, where small and scattered agricultural lands with vegetables grown by local residents were witnessed. These vegetables were growing in polluted soil and thus had potential threats to their consumers. The pollution source might be from the mine tailings upstream. The high level of Cu content could partly be attributed to Cu-rich suspended particles carried by the running water, which deposited and accumulated in this area.

**Figure 6:**
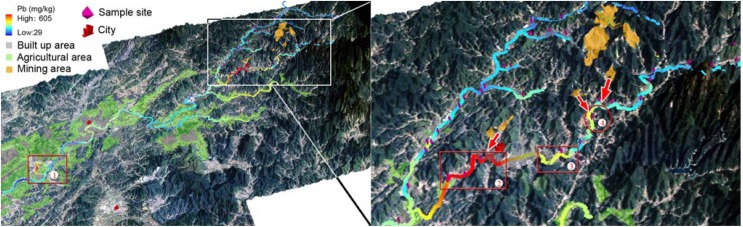
Maps showing the distribution of total Pb content in the soil along the Le’an River and its branches (left), and in the soil around mining area (right).

The left of Figure 6 shows an overall distribution of total Pb content in top soil along the Le’an River and its branches, and the right portion provides details of the total Pb distribution around the Yinshan Lead and Zinc Mine. Four regions with relatively high total Pb content were highlighted by red frame and numbered with “①”, “②”, “③”, and “④”. The concentration of Pb in the region ① reached as high as 230 mg/kg. Considering the high level of Cu content indicated by the same sample collected at this site, further survey should be encouraged to evaluate the soil contamination level in this area.

The spatial distribution of total Pb content at the middle and upper reaches of the Le’an River is shown in Figure 6. The soil samples collected along the Jishui River showed up with generally high values in comparisons with those of along the Le’an River. The Pb content of soil samples from around the Dexing City, which was noted as the region ②, ranged from 202 mg/kg to 605 mg/kg. The Yinshan Lead and Zinc Mine was likely the source. The transportation routes were indicated by an arrow. Similarly to the total Cu distribution along the Jishui River, the regions ③ and ④ both showed up with relatively high total Pb content. 

### 3.4. Watershed Delineation

**Figure 7:**
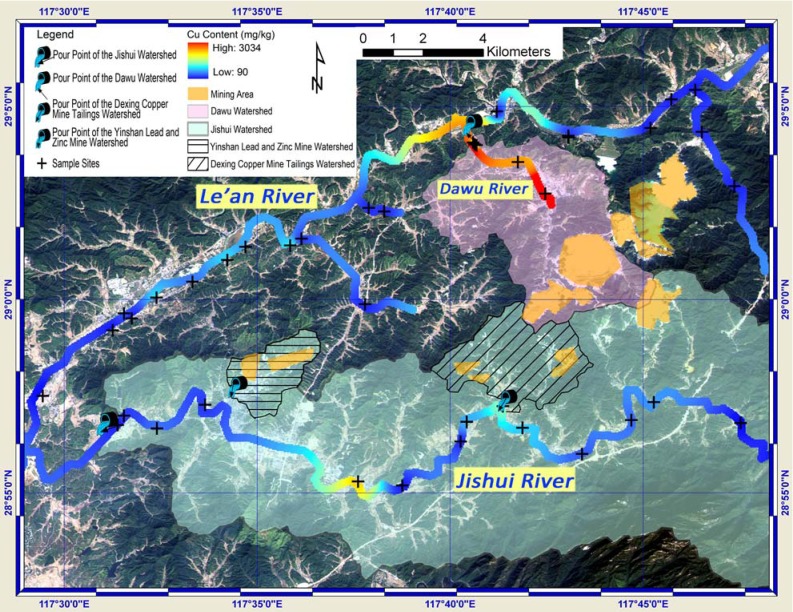
Spatial relationships between watersheds, mining area and total Cu content.

**Figure 8:**
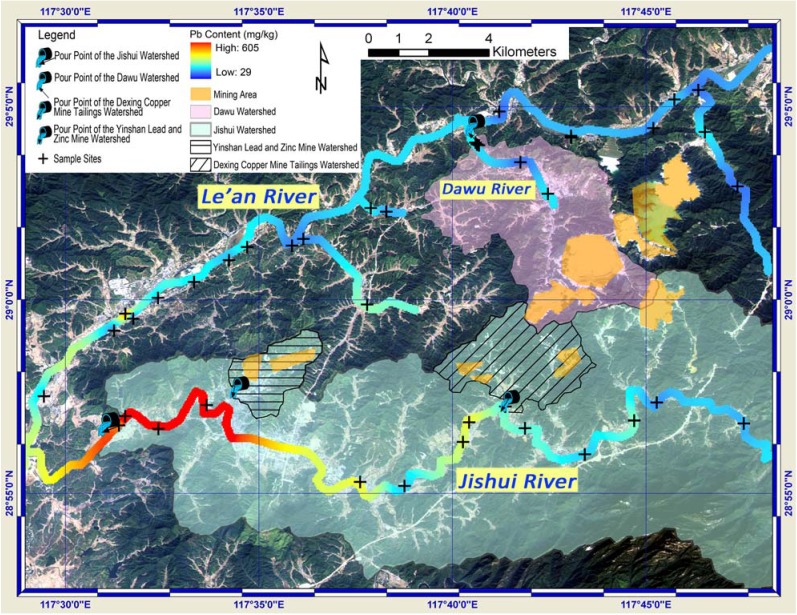
Spatial relationships between watersheds, mining area and total Pb content.

Four watersheds, namely the Jishui watershed, the Dawu watershed, the Dexing Copper Mine Tailings Watershed and the Yinshan Lead and Zinc Mine Water, were extracted and shown with their pour points in Figure 7 and Figure 8. The latter two small watersheds are parts of the Jishui watershed. The direction of the arrow in the symbol of the pour point indicates the water flow direction of specific watershed at the pour point. The ALOS remote sensing image serves as background, from which the land use and land cover can be identified. Coupled with Cu/Pb content along rivers, sample sites and vector data highlighting the mining area, Figure 7 and Figure 8 provide a visual and comprehensive inspection on the spatial relationships between the polluted sites and the sources. The significant role that topography plays in the transportation of the Cu and Pb was further discussed.

The Dawu watershed covers the major part of the Dexing Copper Mine (Figure 7). The soil samples along the Dawu River were observed with high content of Cu, whereas the Pb content in these samples was low. The Cu content in the soil samples collected outside this watershed was generally much lower. This indicates that the mining activities in the Dexing Copper Mine significantly increased the total Cu content in the soil along the Dawu River, which was in the Dawu watershed. The soil sample near the pour point of the Dexing Copper Mine tailings watershed showed up with relatively high value of Cu content. The previously proposed explanation for the relatively high Cu value observed in region ④ (in Section 3.3) can thus be confirmed: Cu element was transported from the two mine tailings.

The Jishui River ran from east to west (Figure 8). The soil samples downstream the Jishui River became to show high value in total Pb content from the pour point of the Yinshan Lead and Zinc Mine watershed. In case of raining, part of rainfall in this watershed became streamflow carrying Pb, and accumulated at the pour point. Considering the high value of Pb content in the samples collected downstream of the pour point, it is feasible to infer that the Yinshan Lead and Zinc Mine was the source of Pb pollution in the soil in this area. As the Dexing City located here, its citizens were in the risk of the Pb pollution in soil. For the soil samples along the Le’an River and its other branches, the Pb content was generally low. This indicates that the topography was a critical aspect that controlled both the extent and direction of the pollutant transportations. The streamflow resulting from rainfall could be the major carrier of Pb pollutants in this region. 

The areas upstream of the pour point of the Yinshan Lead and Zinc Mine watershed showed up with relative high value of total Pb content (areas in colors ranging from red to yellow). This, however, was the results from data interpolation. More samples are needed for a more accurate assessment of Pb contamination here.

## 4. Conclusions

This study integrates heavy metal content data, remote sensing images, topography data, other ancillary data and GIS analyses for mapping the total Cu and Pb contents spatially. With 3D maps generated from multi-source data, the potential sources and transportation routes of Cu and Pb pollutants were inferred through visual inspections and field survey records. The inference was thereafter confirmed by mapping together the watersheds of the Dawu River, Jishui River and the mining area, the Cu/Pb content in soil, and sample sites. The areas in high risk of Cu or Pb contaminations were also suggested. The important roles that topography and streamflow play in the transportation of Cu and Pb pollutant were graphically illustrated. Bearing in mind the idea “better mapping means better and easier understanding”, this study demonstrated the arts of utilizing multi-source data in the mapping of environmental pollution as well as in the understanding of the role that topography plays in pollutant transport. 

## References

[1] Gupta S.K., Vollmer M.K., Krebs R. (1996). The importance of mobile, mobilisable and pseudo total heavy metal fractions in soil for three-level risk assessment and risk management. Sci. Total Environ..

[2] Hooker P.J., Nathanail C.P. (2006). Risk-based characterisation of lead in urban soils. Chem. Geol..

[3] Bien J.D., Meer J.T., Rulkens W.H., Rijnaarts H.H.M. (2004). A GIS-based approach for the long-term prediction of human health risks at contaminated sites. Environ. Model. Assess..

[4] Poggio L., Vrscaj B. (2009). A GIS-based human health risk assessment for urban green space planning—An example from Grugliasco (Italy). Sci. Total Environ..

[5] Xiao H.G., Ji W. (2007). Relating landscape characteristics to non-point source pollution in mine waste-located watersheds using geospatial techniques. J. Environ. Manag..

[6] Zeng F., Xiao H., Zhou W. (2007). Spatial and temporal variations and their source analysis of Copper, Lead and Zinc in river waters and sediments of the Le’an River. Res. Environ. Sci..

[7] Wu A., Yin S., Wang H., Qin W., Qiu G. (2009). Technological assessment of a mining-waste dump at the Dexing copper mine, China, for possible conversion to an in situ bioleaching operation. Bioresour. Technol..

[8] JAXA AVNIR-2 Characteristics. http://www.eorc.jaxa.jp/ALOS/en/about/avnir2.htm.

[9] Wei F., Zheng C., Chen J., Wu Y. (1991). Study on the background contents on 61 elements of soils in China. Chin. J. Environ. Sci..

[10] Teng Y., Ni S., Wang J., Zuo R., Yang J. (2010). A geochemical survey of trace elements in agricultural and non-agricultural topsoil in Dexing area, China. J. Geochem. Explor..

[11] Kitagishi K., Yamane I. (1981). Heavy Metal Pollution in Soils of Japan.

[12] Davies B. (1977). Heavy Metal Pollution of British Agricultural Soils with Special Reference to the Role of Lead and Copper Mining.

[13] Huff L.C., Lovering T. (1976). Migration of lead during oxidation and weathering of lead deposits. Prof. Pap. U.S. Geol. Surv..

[14] Kick H., Burger H., Sommer K. (1980). Gesamthalte an Pb, Zn, Sn, As, Cd, Hg, Cu, Ni, Cr und Co in Landwirtschaftlich und gaertnerisch genutzten Böden Nordhein-Westfalens. Landwirtsch. Forsch..

